# Association between SARS-CoV-2 variants and post COVID-19 condition: findings from a longitudinal cohort study in the Belgian adult population

**DOI:** 10.1186/s12879-023-08787-8

**Published:** 2023-11-08

**Authors:** Huyen Nguyen Thi Khanh, Laura Cornelissen, Diego Castanares-Zapatero, Robby De Pauw, Dieter Van Cauteren, Stefaan Demarest, Sabine Drieskens, Brecht Devleesschauwer, Karin De Ridder, Rana Charafeddine, Pierre Smith

**Affiliations:** 1 Department of Epidemiology and public health, Sciensano, Brussels, Belgium; 2 Faculty of Medicine and Health Sciences, University of Antwerp, Antwerp, Belgium; 3 Belgian Health Care Knowledge Centre (KCE), Brussels, Belgium; 4 Department of Rehabilitation Sciences, Ghent University, Ghent, Belgium; 5 Department of Translational Physiology, Infectiology and Public Health, Ghent University, Ghent, Belgium; 6 Institute of Health and Society (IRSS), Université catholique de Louvain, Brussels, Belgium

**Keywords:** post-COVID-19 condition, SARS-CoV-2 variants, Long COVID

## Abstract

**Background:**

While many studies on the determinants of post-COVID-19 conditions (PCC) have been conducted, little is known about the relationship between SARS-CoV-2 variants and PCC. This study aimed to assess the association between different SARS-CoV-2 variants and the probability of having PCC three months after the infection.

**Methods:**

This study was a longitudinal cohort study conducted between April 2021 and September 2022 in Belgium. In total, 8,238 adults with a confirmed SARS-CoV-2 infection were followed up between the time of their infection and three months later. The primary outcomes were the PCC status three months post infection and seven PCC symptoms categories (neurocognitive, autonomic, gastrointestinal, respiratory, musculoskeletal, anosmia and/or dysgeusia, and other manifestations). The main exposure variable was the type of SARS-CoV-2 variants (i.e. Alpha, Delta, and Omicron), extracted from national surveillance data. The association between the different SARS-CoV-2 variants and PCC as well as PCC symptoms categories was assessed using multivariable logistic regression.

**Results:**

The proportion of PCC among participants infected during the Alpha, Delta, and Omicron-dominant periods was significantly different and respectively 50%, 50%, and 37%. Participants infected during the Alpha- and Delta-dominant periods had a significantly higher odds of having PCC than those infected during the Omicron-dominant period (OR = 1.61, 95% confidence interval [CI] = 1.33–1.96 and OR = 1.73, 95%CI = 1.54–1.93, respectively). Participants infected during the Alpha and Delta-dominant periods were more likely to report neurocognitive, respiratory, and anosmia/dysgeusia symptoms of PCC.

**Conclusions:**

People infected during the Alpha- and Delta-dominant periods had a higher probability of having PCC three months after infection than those infected during the Omicron-dominant period. The lower probability of PCC with the Omicron variant must also be interpreted in absolute figures. Indeed, the number of infections with the Omicron variant being higher than with the Alpha and Delta variants, it is possible that the overall prevalence of PCC in the population increases, even if the probability of having a PCC decreases.

**Supplementary Information:**

The online version contains supplementary material available at 10.1186/s12879-023-08787-8.

## Background

In October 2021, the World Health Organization (WHO) defined long COVID as post COVID-19 condition (PCC) that occur in COVID-19 patients usually three months after the initial infection with symptoms that last at least 2 months and cannot be explained by an alternative diagnosis. PCC symptoms and their severity may fluctuate over time [1]. PCC is a multisystem disease with a wide range of symptoms, including but not limited to disorders of the respiratory, cardiovascular, musculoskeletal, dermatological, and neurological systems [2, 3]. The mechanisms underlying its pathophysiology are still under debate in the scientific community. On the one hand, PCC can be related to organ damage following an acute SARS-CoV-2 infection such as myocardial infection or renal failure, leading to persistent symptoms [4]. On the other hand, it can be related to a prolonged pro-inflammatory response related to a SARS-CoV-2 infection inducing a dysregulated response of the immune system and mast cells, and persistent symptoms [5]. The most common symptoms of PCC are cognitive and mental health issues, pain, headache, fatigue, anosmia/dysgeusia, neuropathy, dyspnea, cough and chest pain [3, 6, 7]. A systematic review by the European Centre for Disease Prevention and Control found that PCC symptoms were estimated at 51% in community cohorts, but this varies due to differing study designs, emphasizing the need for careful interpretation. For example, a prospective, population-based, observational cohort study in the Netherlands study reported a lower figure of around 12.7% of PCC in the general population infected by SARS-CoV-2 [8]. With over a hundred million SARS-CoV-2 infected people worldwide, PCC could potentially yield a huge burden to individuals, the society and healthcare systems.

Since different SARS-CoV-2 variants may have different associations with the incidence, symptoms and severity of PCC, they might also have a different influence on its pathophysiology [9]. Prior studies have investigated the relationship between SARS-CoV-2 variants and PCC, yielding varied findings. Antonelli et al. [10] and Pontus Hedberg [11] found that people infected by Omicron BA.1 had a lower probability to have PCC than those infected by the ancestral virus, Delta and Alpha variants. Karin Magnuson et al. discovered that there was no discernible difference in the risk of PCC between the Omicron and Delta variants from 14 up to 126 days after testing positive. Nevertheless, at ≥ 90 days post-positive test, individuals infected with Omicron exhibited a lower risk of PCC compared to those infected with Delta [12]. Tala Ballouz at al reported that vaccinated individuals with Omicron had significantly lower odds of developing PCC and similar odds for non-vaccinated PCC with Delta or Omicron compared to non-vaccinated ancestral virus individuals [13]. Since SARS-CoV-2 has continuously mutated, studying the association of SARS-CoV-2 variants and PCC is essential to improve the understanding of the pathophysiology and risk factors for PCC. In Belgium, a longitudinal cohort study (COVIMPACT) was set up in April 2021 to follow up individuals with a recent SARS-CoV-2 infection and to assess the occurrence of PCC [14].This study aims to assess the association between different SARS-CoV-2 variants and the probability of having PCC three months after the infection, using data from the COVIMPACT study.

## Methods

### Study design and population

**Fig. 1:**
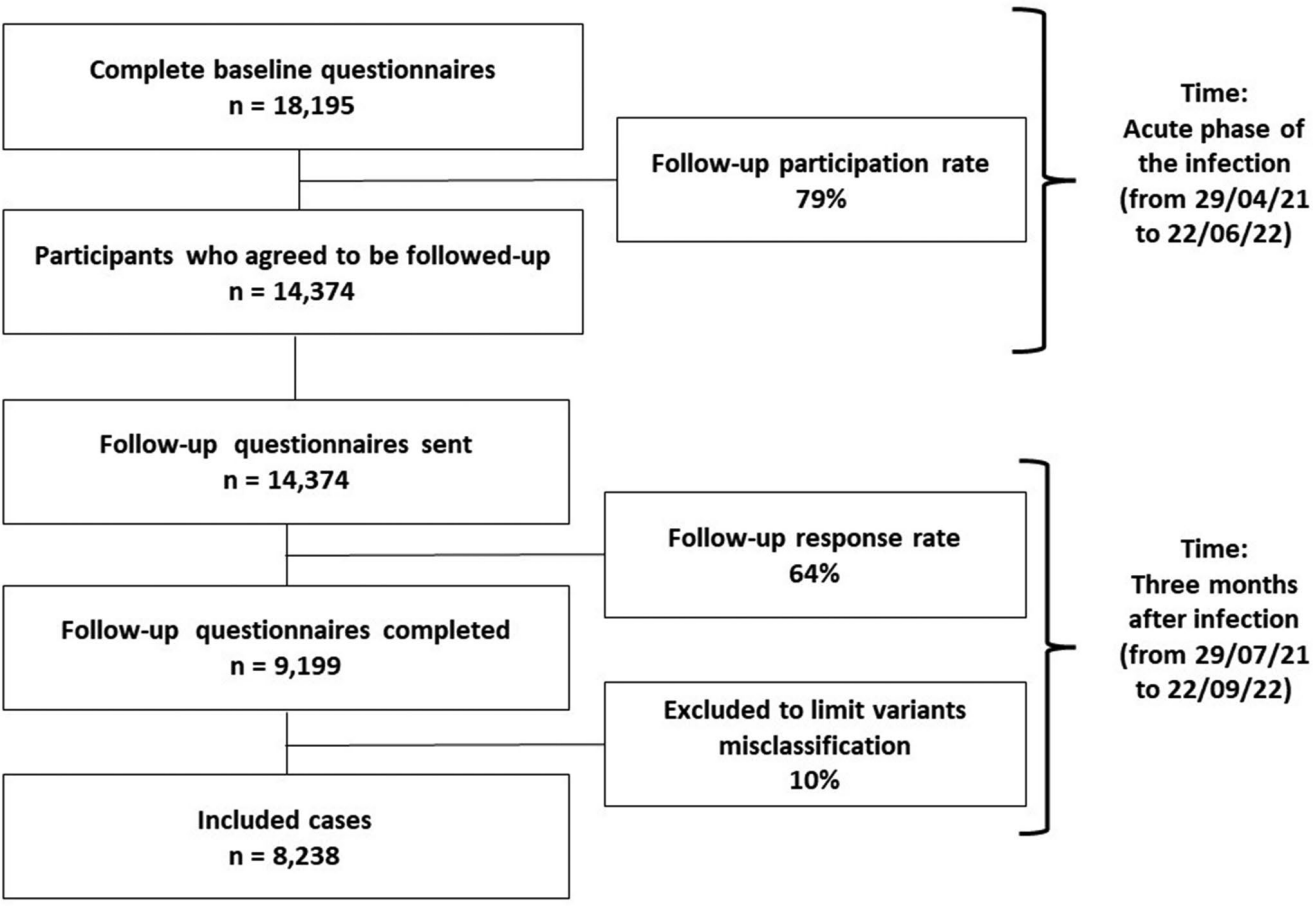
Flow diagram

This study used longitudinal data from COVIMPACT cohort study conducted in Belgium [14]. People aged 18 years and older, living in Belgium, with a recent SARS-CoV-2 infection confirmed via a molecular or an antigen test were contacted for tracing purposes by contact tracing call centers [15, 16]. Participants completed two online questionnaires: a baseline questionnaire sent at the time of their confirmed SARS-CoV-2 infection (i.e. after the call from the contact tracing center) and a follow-up questionnaire sent three months later. The baseline questionnaires were sent between April 29, 2021 and June 22nd, 2022, and the follow-up questionnaires between July 29, 2021 and September 22nd, 2022. The published study protocol [14] showed that 5% of all Belgian adults infected during the study period completed the baseline questionnaire. The flow diagram of the present study is presented in Fig. 1. In total, 79% of participants who completed the baseline questionnaire agreed to participate in the follow-up, and 64% of participants who received the follow-up questionnaire completed it. In total, 9,199 individuals completed both questionnaires. However, to limit misclassification of SARS-CoV-2 variants (see Exposure assessment), only 8,238 participants were included in this study.

### Measurement

#### Outcome assessment

The primary outcome of this study was the PCC status of participants, i.e. whether or not they self-reported at least one symptom of PCC three months after the infection (binary variable). PCC was assessed in the 3-months follow-up questionnaire, based on the definition form the National Institute for Health and Care Excellence (NICE): “signs and symptoms that develop during or after an infection consistent with COVID-19, continue for more than twelve weeks and are not explained by an alternative diagnosis” [17]. The question asked was: *“Within the last seven days have you had any of these symptoms, that you did not experience before onset of your COVID-19 illness*?”. Thirty potential PCC symptoms were listed, based on published guidelines of the WHO and the NICE [1, 17].

The second outcome of this study was PCC symptoms categories. Based on the classification proposed by Fernandez de las Peñas et al., the thirty potential PCC symptoms collected in this study can be classified as follows [18]: [1] Neurocognitive post-COVID (*sleeping problems, headache, memory problems, dizziness, confusion, problems speaking, incontinence, seizures*), [2] autonomic post-COVID (*chest pain, palpitations*), [3] gastrointestinal post-COVID (*constipation/diarrhea, stomach pain, nausea/vomiting*), [4] respiratory post-COVID (*general fatigue, dyspnea, persistent cough*), [5] musculoskeletal post-COVID (*muscle pain, joint pain, swelling-oedema*), [6] anosmia and/or dysgeusia post-COVID (*loss of smell, loss of taste)*, [7] other manifestations (*tingling feeling, loss of appetite, problem seeing, ringing in ears, general malaise, weight loss, skin rashes, problem swallowing, other symptom(s)*). Each symptom category was a binary variable (having or not these symptoms) and as people with PCC tend to report numerous and heterogeneous symptoms, a participant can be in more than one category.

#### Exposure assessment

The exposure variable in this study was the type of SARS-CoV-2 variants and subvariants, which was an indicator at the population level extracted from the SARS-CoV-2 surveillance system of Sciensano, the Belgian institute for health [19]. In this surveillance system, at least 5% of all positive RT-PCR samples in Belgium was randomly selected for sequencing [20], which met the recommendation from the European Centre for Disease Prevention and Control (ECDC) [21]. Since 5 April 2021 until 17 September 2022, the Belgium COVID-19 Epidemiological Situation Dashboard of Sciensano reported daily percentages of eight SARS-CoV-2 (sub)variants including Alpha, Beta, Gamma, Delta, Omicron, Omicron BA.2, Omicron BA.4 and Omicron BA.5 [19].

Each study day, the dominant variant (i.e. responsible for more than 80% of daily infections) was selected and assigned to participants who reported testing positive for SARS-CoV-2 on the same day. The threshold of 80% was chosen to limit the misclassification of the exposure variable due to periods with concomitant variants [22]. Consequently, the days for which no variant was above the 80% threshold were not included in the analysis (e.g. a day when the Alpha variant was responsible for 50% of infections and the Delta the other 50%). The analyses were therefore carried out on 407 days out of the 448 of the study periods (91%) and on 8238 participants out of the 9199 (90%). Sensitivity analyses were conducted with a 70% and 90% threshold (supplementary Table 1). Additional sensitivity analyses were also performed to compare the profile of excluded and included cases (supplementary Table 2).

#### Other covariates

Covariates including demographic factors, the presence of comorbidities, vaccination status before COVID-19 (completed one, two, or three doses), and the number of COVID-19 acute symptoms were collected in the baseline questionnaire as they may be associated with the probability of having PCC [2]. As the variable on the number of acute COVID-19 symptoms has a positive skewed distribution, we classified it into four categories (none, 1–4, 5–8, > 8) using regression tree analysis with the variable as both dependent and independent variable. The regression tree analysis is an appropriate method for identifying cut-off points that represent the distribution of the variable in the sample and it contributes to detect possible non-linear trends and risk groups. The presence of comorbidities was assessed with the following question: “Before your COVID-19 infection, have you had any of the following diseases or conditions?” and using the following list of chronic diseases: asthma, chronic bronchitis, chronic obstructive pulmonary disease, emphysema, high blood pressure (hypertension), chronic cardiovascular disease (excluding high blood pressure), diabetes mellitus, type 1 of 2, chronic neurological disorder; chronic kidney disease (including dialysis), chronic liver disease, cancer (excluding blood cancer, e.g. leukaemia, lymphoma), blood cancer (e.g. leukaemia, lymphoma), diseases or treatments that suppress the immune system (except HIV), having a transplantation or on the waiting list for a transplantation, other long-term illness (please specify).

### Statistical analysis

All analyses were carried out using the statistical package R version 4.1.0 [23]. The number of missing data among each variable is reported in Table 1. These missing values account for considerably less than 10% of the total observations, hence we opted for a complete case analysis [24]. Descriptive statistics were performed to show the distribution of variables. Categorical variables were summarized by the frequencies and percentages of the levels, while numeric variables were summarized by their mean and standard deviation or median and IQR (inter-quartile range) based on their distribution. Chi-square tests were used to test the difference of the distribution of PCC status (% having or not PCC) in the different explanatory variables.

**Table 1:** General characteristics of study population and distribution of Post COVID-19 Condition

Characteristic	Total	Having post COVID-19 condition	p-value**
	N = 8,238*	N = 3,709 (45.0%) (% by row)	
Variant			< 0.001
Alpha	1,135 (13.78%)	573 (50.48%)	
Delta	3,927 (47.67%)	1,969 (50.14%)	
Omicron	3,176 (38.55%)	1,167 (36.74%)	
Gender			< 0.001
Male	2,816 (34.56%)	1,035 (36.75%)	
Female	5,331 (65.44%)	2,633 (49.39%)	
Missing data	91	41	
Age			< 0.001
18–25	680 (8.91%)	314 (46.18%)	
26–45	3,381 (44.29%)	1,593 (47.12%)	
46–65	3,118 (40.84%)	1,388 (44.52%)	
66+	455 (5.96%)	139 (30.55%)	
Missing data	604	275	
Education			< 0.001
Secondary school or below	2,173 (26.74%)	1,083 (49.84%)	
Higher education	5,953 (73.26%)	2,563 (43.05%)	
Missing data	112	63	
Having chronic disease (Yes)			< 0.001
No	7,620 (92.62%)	3,337 (43.79%)	
Yes	607 (7.38%)	370 (60.96%)	
Missing data	11	2	
Body Mass Index (BMI)			< 0.001
Normal (BMI 18.5–24.9)	3,596 (43.65%)	1,542 (42.88%)	
Overweight (BMI 25.0-29.9)	2,988 (36.27%)	1,334 (44.65%)	
Obesity (BMI 30.0+)	1,654 (20.08%)	833 (50.36%)	
Hospitalization (Yes)			< 0.001
No	8,061 (98.96%)	3,613 (44.82%)	
Yes	85 (1.04%)	60 (70.59%)	
Missing data	92	36	
COVID-19 vaccination status			< 0.001
None	483 (5.95%)	199 (41.20%)	
Partial	99 (1.22%)	55 (55.56%)	
Complete primary schedule	2,396 (29.54%)	1,210 (50.50%)	
Complete primary schedule and booster	5,133 (63.28%)	2,180 (42.47%)	
Missing data	127	65	
Number of Covid-19 symptoms at baseline			< 0.001
None	370 (4.49%)	91 (24.59%)	
1–4	2,512 (30.49%)	826 (32.88%)	
5–8	3,236 (39.28%)	1,429 (44.16%)	
> 8	2,120 (25.73%)	1,363 (64.29%)	
Post COVID-19 condition			
Neurocognitive symptoms, yes	1,975 (23.97%)	/	
Autonomic symptoms, yes	556 (6.75%)	/	
Gastrointestinal symptoms, yes	629 (7.64%)	/	
Respiratory symptoms, yes	2,217 (26.91%)	/	
Musculoskeletal symptoms, yes	1,089 (13.22%)	/	
Anosmia and/or dysgeusia, yes	666 (8.08%)	/	
Other manifestations, yes	1,376 (16.70%)	/	

*Percentage by column **Pearson’s Chi-squared test

Multivariable logistic regression was used to assess the association between the different SARS-CoV-2 variants and PCC. The final model was developed based on three steps. First, a multivariable logistic regression model was used, which included all covariates as fixed effects. As all covariates were significantly associated with PCC, they were kept to the next step. Second, due to the association between SARS-CoV-2 variants and the severity of COVID-19 acute infection [25], interactions between SARS-CoV-2 variants and covariates related to the acute infection (i.e. number of COVID-19 acute symptoms and hospitalization status) were tested, but turned out to be not significant. Finally, we performed the Hosmer-Lemeshow goodness of fit test to determine whether the fitted model adequately described the outcome in the data (p = 0.27). The p-value was above alpha = 0.05 (accepted H0), so the null hypothesis could not be rejected, meaning this model predicted the outcome well. Sensitivity analyses with a 70% and 90% threshold (see supplementary material Table 1) confirmed the result from the multivariable logistic regression model. Since there is evidence suggesting a potential interaction between variants and vaccination [13], we conducted a sensitivity analysis to investigate the combined impact of SARS-CoV-2 variants and COVID-19 vaccination status prior to infection (both vaccinated and non-vaccinated groups). For detailed results, please refer to supplementary material Tables 3 and 4.

To investigate a group of predictors that can be used to make accurate predictions for PCC, the decision tree method was performed using the rpart package and a chi-squared automatic interaction detector, with 80% participants as the training dataset, and the remaining 20% as the test dataset. The training dataset was used to build decision tree, and the test dataset was used to validate the prediction. The measure used to split nodes was the Gini index, and pruning was used to avoid overfitting the model. Table 2 shows the variable assignments utilized in both the logistic regression analysis and the decision tree model.

**Table 2:** Bivariate and Multivariable model of Post COVID-19 Condition status which was adjusted for sex, age, education, Body Mass Index (BMI), having a chronic disease, number of Covid-19 acute symptoms, COVID-19 vaccination status, hospitalization status

Characteristic	Bivariate			Multivariable		
	Odds Ratio	95% Confidence Interval	p-value	Odds Ratio	95% Confidence Interval	p-value
Variant						
Omicron	REF	REF	REF	REF	REF	REF
Alpha	1.76	1.53–2.01	< 0.001	1.61	1.33–1.96	< 0.001
Delta	1.73	1.57–1.90	< 0.001	1.73	1.54–1.93	< 0.001
Gender						
Male	REF	REF	REF	REF	REF	REF
Female	1.68	1.53–1.84	< 0.001	1.57	1.41–1.75	< 0.001
Age						
18–25	REF	REF	REF	REF	REF	REF
26–45	1.04	0.88–1.23	0.731	1.01	0.84–1.21	0.635
46–65	0.94	0.79–1.10	0.426	0.96	0.80–1.16	0.785
66+	0.51	0.40–0.66	< 0.001	0.66	0.50–0.87	0.003
Education						
Secondary school or below	REF	REF	REF	REF	REF	REF
Higher education	0.76	0.69–0.84	< 0.001	0.77	0.69–0.86	< 0.001
Body Mass Index (BMI)						
Normal (BMI 18.5–24.9)	REF	REF	REF	REF	REF	REF
Overweight (BMI 25.0-29.9)	1.07	0.97–1.18	0.233	1.12	1.00-1.25	0.050
Obesity (BMI 30.0+)	1.35	1.20–1.52	< 0.001	1.38	1.21–1.58	< 0.001
Having chronic disease						
No	REF	REF	REF	REF	REF	REF
Yes	2.00	1.69–2.38	< 0.001	1.61	1.33–1.96	< 0.001
Number of Covid-19 symptoms at baseline						
None	REF	REF	REF	REF	REF	REF
1–4	1.50	1.17–1.94	0.001	1.86	1.41–2.49	< 0.001
5–8	2.42	1.90–3.12	< 0.001	2.94	2.24–3.91	< 0.001
> 8	5.52	4.30–7.14	< 0.001	5.99	4.53–8.02	< 0.001
COVID-19 vaccination status						
None	REF	REF	REF	REF	REF	REF
Partial	1.78	1.16–2.77	0.009	1.74	1.06–2.88	0.030
Complete primary schedule	1.46	1.20–1.78	< 0.001	1.74	1.38–2.20	< 0.001
Complete primary schedule and booster	1.05	0.87–1.27	0.656	1.57	1.25–1.96	< 0.001
Hospitalization						
No	REF	REF	REF	REF	REF	REF
Yes	2.95	1.87–4.80	< 0.001	2.25	1.35–3.84	0.002

The difference in the distribution of the seven groups of PCC symptoms between the different SARS-CoV-2 variants was described by using frequencies and percentages and tested using Pearson’s Chi-squared test. Seven multivariable logistic regression models were performed to assess the association between the different SARS-CoV-2 variants and PCC symptom categories with the same approach as with the model on PCC. The Hosmer-Lemeshow goodness of fit test was used for seven models and no significant p-values were reported, meaning that these models well predicted the outcome.

## Results

The general characteristics of the participants and distribution of PCC are shown in Table 1. Most participants (47.7%) were included during the Delta-dominant period, followed by the Omicron-dominant period (38.5%), and the Alpha-dominant period (13.8%). In the total sample, nearly half (45.0%) of the participants reported having PCC. While there was no difference in the proportion of participants reporting PCC during the Alpha- and Delta-dominant periods (50.5% vs. 50.1% respectively), fewer participants reported having PCC during the Omicron-dominant period (36.7%, p < 0.001). Respiratory and neurocognitive PCC symptoms made up more than half of all cases (26.9% and 24.0% respectively) and were the most prevalent symptom groups for PCC, followed by other manifestations (16.7%), musculoskeletal symptoms (13.2%), anosmia and/or dysgeusia (8.1%), gastrointestinal symptoms (7.6%), and autonomic symptoms (6.7%).

Regarding the other participant characteristics, the proportion of PCC was significantly higher among women than men (49.4% vs. 36.7%, p < 0.001). The younger groups (18–25 years old, 26–45 years old, 46–64 years old) had a significantly higher proportion of PCC than the older (over 65 years old) (46.2%, 47.1%, 44.7% vs. 30.5% respectively, p < 0.001). A statistically significant higher proportion of PCC was also observed among participants hospitalized following COVID-19 (70.6%), those with a higher number of acute COVID-19 symptoms (> 8 symptoms, 64.3%), with obesity (50.4%), with a lower educational level (49.8%), and being partially vaccinated for COVID-19 (55.6%).

The results of bivariate and multivariable logistic regression models with the PCC status as the outcome are presented in Table 2. The final multivariable model showed that participants infected during the Alpha-dominant period and Delta-dominant period had a significantly higher odds of having PCC than those infected during the Omicron-dominant period (OR = 1.61, 95%CI = 1.33–1.96 and OR = 1.73, 95%CI = 1.54–1.93 respectively). Regarding the other variables, female sex, obesity, chronic disease, being vaccinated for COVID-19, having more than one acute COVID-19 symptom, and hospitalization following COVID-19 were significantly associated with an increased odd of PCC, while being 65 years or older, and having a higher educational level were associated with a decreased odds of PCC. The sensitivity analysis of joint association with vaccination and variants in Supplementary Table 4 also showed that vaccinated participants infected during the Alpha-dominant period and Delta-dominant period had a significantly higher odds of having PCC than those infected during the Omicron-dominant period.

**Fig. 2:**
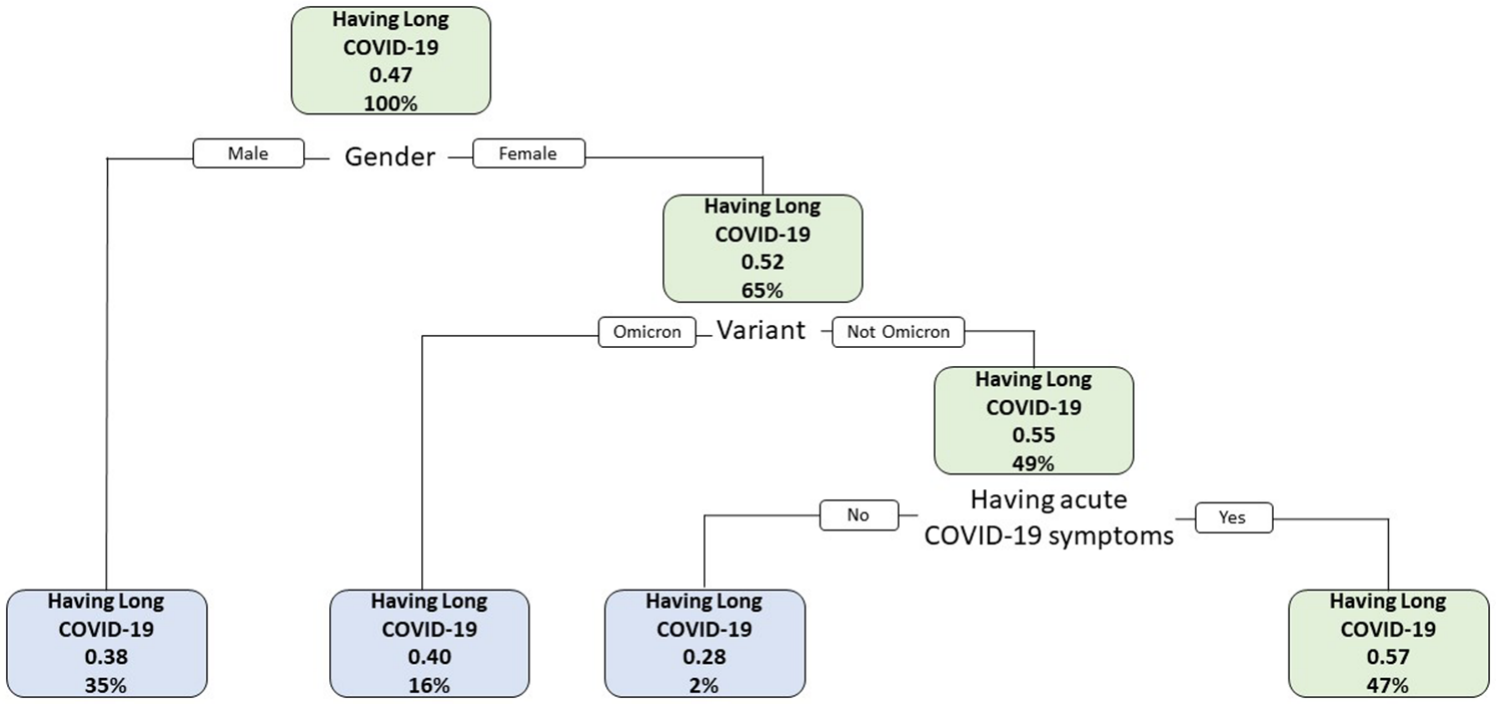
Decision tree model for Post COVID-19 Condition

As shown in Fig. 2, the importance of variables in the decision tree model was presented as a root-to-leaf structure, with gender being the first variable or root node, followed by variant types and having acute COVID-19 symptoms, in order of importance. The group with the higher proportion of PCC (57%) was women, not infected with Omicron (infected with the Alpha or Delta variant) and having at least one acute COVID-19 symptom, accounting for 47% of the total sample, which aligned with the result of regression modeling in Table 2. The predictive accuracy of the decision tree model was 63.51%. The test dataset included 1450 subjects, of whom 921 persons had their true PCC status correctly predicted by the prediction model.

**Fig. 3:**
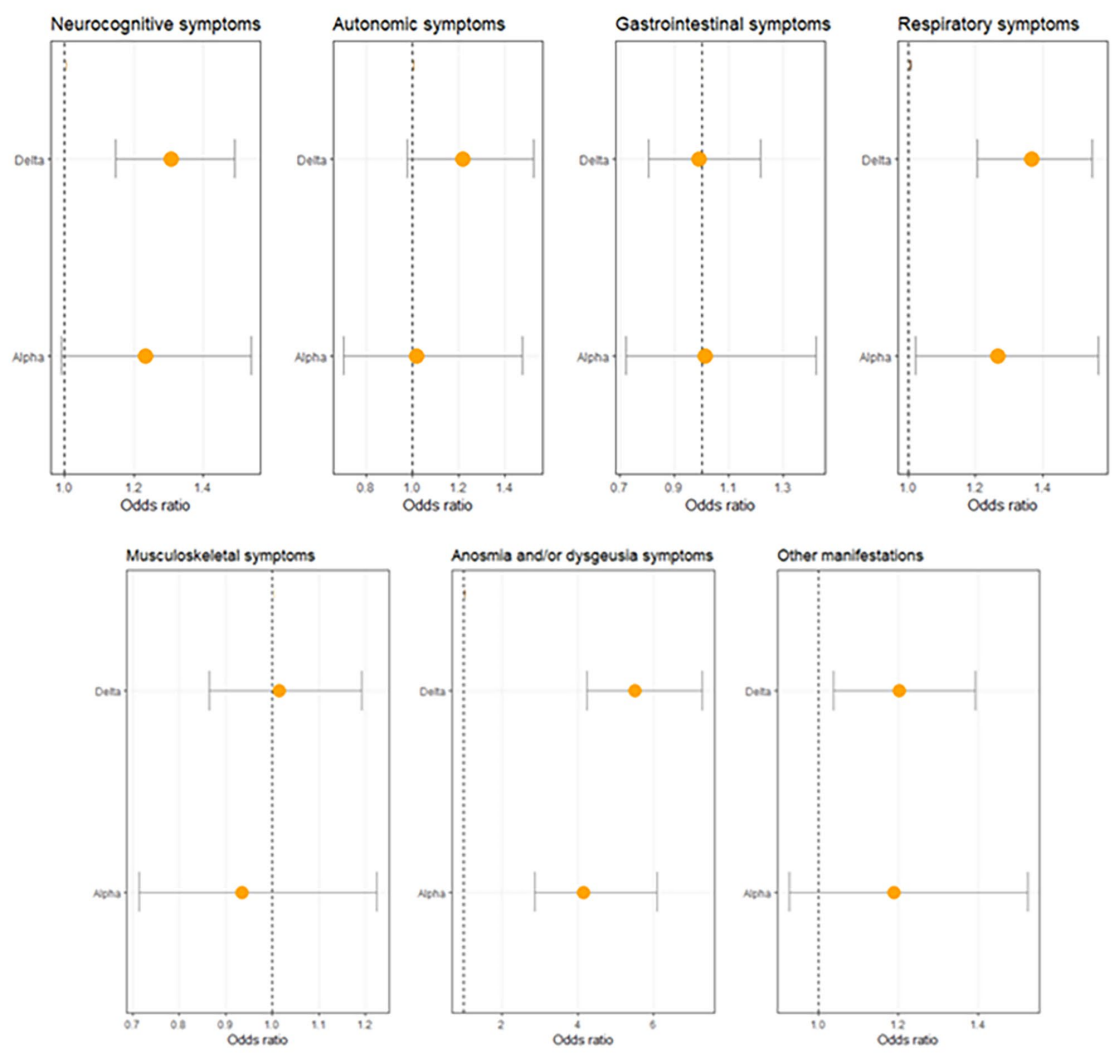
Associations between Post COVID-19 condition symptom groups and SARS-CoV-2 variants (reference = Omicron) adjusted by age, gender, education level, body-mass index, number of COVID-19 symptoms at baseline, presence of comorbidities, and hospitalization status

The distribution of the seven groups of PCC symptoms between the different SARS-CoV-2 variants is shown in Table 3 and modeled in Fig. 3. Participants infected during the Alpha and Delta -dominant periods were significantly more likely to experience anosmia and/or dysgeusia (respectively 10%, OR = 4.15 and 12%, OR = 5.51) and respiratory symptoms (respectively 31%, OR = 1.26, and 30%, OR = 1.36) compared to those infected during Omicron-dominant period (2.5% and 22%). Participants infected during Delta-dominant periods were also significantly more likely to experience neurocognitive symptoms (28%, OR = 1.31) and other manifestations (19%, OR = 1.21) compared to those infected during Omicron-dominant period (20% and 14%).

**Table 3:** The distribution of SARS-CoV-2 variants among Post COVID-19 Condition symptoms

Characteristic	Alpha, N = 1,135	Delta, N = 3,797	Omicron, N = 1530
Neurocognitive symptoms, yes	314 (28%)	1,030 (26%)	631 (20%)
Autonomic symptoms, yes	84 (7.4%)	299 (7.6%)	173 (5.4%)
Gastrointestinal symptoms, yes	102 (9.0%)	304 (7.7%)	223 (7.0%)
Respiratory symptoms, yes	351 (31%)	1,161 (30%)	705 (22%)
Musculoskeletal symptoms, yes	170 (15%)	534 (14%)	385 (12%)
Anosmia and/or dysgeusia, yes	110 (9.7%)	478 (12%)	78 (2.5%)
Other manifestations, yes	213 (19%)	703 (18%)	460 (14%)

## Discussion

This study aimed to assess the association between different SARS-CoV-2 variants and the probability of having PCC three months after the infection in a sample of the adult population in Belgium infected with SARS-CoV-2 between April 2021 and September 2022.

This study revealed that participants infected during the Alpha- and Delta-dominant periods had a significantly higher odds of having PCC than those infected during the Omicron-dominant period. This may encompass a lessened burden on healthcare resources, and potentially decreased long-term healthcare expenses associated with managing PCC. However, the lower odds of PCC with Omicron must be interpreted in absolute figures and according to the evolution of the number of infections. Indeed, the number of infections with the Omicron variant being higher than with the Alpha and Delta variants, it is possible that the overall prevalence of PCC in the population increases, even if the probability of having a PCC decreases. So far, the association between SARS-CoV-2 variants and PCC has been little studied. One study in the UK published in June 2022 [10] confirms this result and found that people infected with Omicron were less likely to experience PCC than those infected with the Delta variant. These results suggest that the risk of PCC is higher for the Alpha and Delta variant than for Omicron even taking into account factors such as the vaccination status, the number of symptoms in the acute phase of the infection, the hospitalization status, and several sociodemographic characteristics. Therefore, the results of the present study suggest that Omicron may be inherently less virulent, both in terms of acute and prolonged pathophysiology, than the Alpha and Delta variants. Although not related to the primary research question, this study also found that people vaccinated for COVID-19 had a higher odds of PCC, while other studies have shown that vaccination as well as a longer time after vaccination decreased the odds of PCC [10]. This result is probably linked to the timing of vaccination in Belgium: people at risk in the acute phase of COVID-19 were vaccinated as a priority, and these same individuals may also be at greater risk of developing PCC.

The present study also found that participants infected during the Alpha- and Delta-dominant periods were more likely to experience respiratory and anosmia/dysgeusia symptoms of PCC than those infected during the Omicron-dominant period, taking into account other covariates such as the number of acute COVID-19 symptoms and the vaccination status. Other studies found that respiratory symptoms, including shortness of breath and cough, were common in individuals with PCC [4] and were associated with previous infection with the Alpha and Delta variants [26]. For example, one study found a common cluster of cardiorespiratory symptoms of PCC among people infected with the Alpha and Delta variants [27]. To date, few studies have been carried out on the association between Omicron infections and respiratory symptoms of PCC in comparison to the other variants. One study found that the odds of PCC was lower among people infected with the Omicron variant in comparison the ancestral virus and Delta variants, but found no significant difference in the prevalence of symptom clusters between the different variants [13]. The present study also found that participants infected during the Delta-dominant period were more likely to report neurocognitive and anosmia/dysgeusia symptoms of PCC in comparison to those infected during the Omicron-dominant period. Anosmia was confirmed as an important neurologic symptom of PCC and associated with potential COVID-19 neurotropism [28]. People with PCC exhibit anosmia after SARS-CoV-2 infection because the virus enters the body through the olfactory epithelium and the expression of TMPRSS2 is upregulated in response to ACE2 [28]. Our results suggest that this mechanism may be more important for the Alpha and Delta variants than for the Omicron variant. César Fernández et al. also found that patients infected with the Alpha or Delta variants reported more symptoms at anosmia or dysgeusia than those infected with the ancestral virus (p < 0.01) [29].

This study highlights the importance of continuous monitoring of the different SARS-CoV-2 variants and their health impact because they can influence the overall risk and symptoms of PCC, and potentially provide elements to better understand its mechanisms and pathophysiology. The main strengths of this study were its [1] consistent data collection over time, [2] extensive list of self-reported PCC symptoms, [4] large sample size, and [5] long study period, allowing us to assess the association between three SARS-CoV-2 variants and PCC and its different symptoms. However, this study also has several limitations. The main limitation is the selection bias due to the design of the study, which may limit the generalizability of results beyond the study population. As previously explained, the study protocol [14] showed that the proportion of people between 46 and 65 years, of women, and of people reporting at least one acute COVID-19 symptom was higher among cohort participants than in the eligible population, resulting in initial sample selection bias. However, no information was available on the proportion of PCC in the eligible population, the proportion of PCC in this study may be underestimated (e.g. people with PCC may not be in good enough condition to respond to the survey) or overestimated (e.g. people without persistent symptoms may place less emphasis on completing the survey) due to study design. The proportion of PCC being higher in women and in people who have had acute symptoms of COVID-19, it is more likely that selection bias induces an overestimation of the proportion of PCC. Second, different periods of variant exposure were defined using national data and cases were included based on their time of infection, with a risk of misclassification of the type of variants at the individual level, especially during periods with concomitant variants. To limit the misclassification bias, a 80% threshold was used, and cases were included only if infected on a day with a single variant responsible for more than 80% of daily infection. Sensitivity analyses were performed with a 70% and 90% threshold (supplementary Table 1) with similar results, but no Alpha cases with the 90% threshold. The use of a threshold and the exclusion of certain cases can also lead to a selection bias. Sensitivity analyzes presented in Supplementary Table 2 showed that the proportion of PCC was slightly higher among excluded cases than included cases (50% versus 45%), thus underestimating the proportion of PCC in the study (45.5% in the total number of cases). Third, PCC symptoms were self-reported and without a control group. However, some symptoms are common to many other diseases and infections that affect the general population, which may result in an overestimation of PCC in our study. Furthermore, although participants reported not having these symptoms before their SARS-CoV-2 infection, we cannot verify that the symptoms are not explained by an alternative diagnosis, which may also lead to an overestimation of the proportion of PCC. Finally, no information was collected on SARS-CoV-2 reinfection during the follow-up of individuals, which may be an important confounding factor.

## Conclusion

This study found that people infected with the Alpha and Delta variants had a significantly higher probability of having PCC three months after their SARS-CoV-2 infection than those infected with Omicron. The lower probability of PCC with the Omicron variant must be interpreted in absolute figures. Indeed, the number of infections with the Omicron variant being higher than with the Alpha and Delta variants, it is possible that the overall prevalence of PCC in the population increases, even if the probability of having a PCC decrease. This study also found that participants infected with the Alpha and Delta variants were more likely to report neurocognitive, respiratory, and anosmia/dysgeusia as symptoms of PCC. This result highlights the importance of monitoring the health impact of the different SARS-CoV-2 variants as it may provide elements to better understand the mechanisms and pathophysiology of PCC. Future research should assess the effects of SARS-CoV-2 variants on PCC but based on direct testing of the variants and with a control group, in order to limit misclassification bias.

### Electronic supplementary material

Below is the link to the electronic supplementary material.

Supplementary Material 1

Supplementary Material 2

Supplementary Material 3

Supplementary Material 4

## Data Availability

The data of this study are available from the corresponding author upon reasonable request.

## List of abbreviations

ECDC: European Centre for Disease Prevention and Control
ICU: Intensive Care Unit
NICE: National Institute for Health and Care Excellence
OR: Odd Ratio
PCC: Post COVID-19 Condition
WHO: World Health Organization

## References

[1] World Health Organization. A clinical case definition of post COVID-19 condition by a Delphi consensus, 6 October 2021 October 2021 [Available from: https://www.who.int/publications/i/item/WHO-2019-nCoV-Post_COVID-19_condition-Clinical_case_definition-2021.1.

[2] Akbarialiabad H, Taghrir MH, Abdollahi A, Ghahramani N, Kumar M, Paydar S (2021). Long COVID, a comprehensive systematic scoping review. Infection.

[3] Castanares-Zapatero D, Chalon P, Kohn L, Dauvrin M, Detollenaere J, de Maertens C (2022). Pathophysiology and mechanism of long COVID: a comprehensive review. Ann Med.

[4] Michelen M, Manoharan L, Elkheir N, Cheng V, Dagens A, Hastie C (2021). Characterising long COVID: a living systematic review. BMJ Global Health.

[5] Afrin LB, Weinstock LB, Molderings GJ (2020). Covid-19 hyperinflammation and post-covid-19 Illness may be rooted in mast cell activation syndrome. Int J Infect Dis.

[6] Han Q, Zheng B, Daines L, Sheikh A. Long-term sequelae of COVID-19: a systematic review and Meta-analysis of one-year Follow-Up studies on Post-COVID symptoms. Pathogens. 2022;11(2). 10.3390/pathogens11020269 PMC8875269 35215212

[7] Almas T, Malik J, Alsubai AK, Jawad Zaidi SM, Iqbal R, Khan K et al. Post-acute COVID-19 syndrome and its prolonged effects: an updated systematic review. Ann Med Surg (Lond). 2022:103995. 10.1016/j.amsu.2022.103995 PMC9197790 35721785

[8] European Centre for Disease Prevention and Control (ECDC). Prevalence of post COVID-19 condition symptoms: a systematic review and meta-analysis of cohort study data, stratified by recruitment setting 31 Oct 2022 [Available from: https://www.ecdc.europa.eu/en/publications-data/prevalence-post-covid-19-condition-symptoms-systematic-review-and-meta-analysis.

[9] Meurisse M, Van Oyen H, Blot K, Catteau L, Serrien B, Klamer S (2022). Evaluating methodological approaches to assess the severity of Infection with SARS-CoV-2 variants: scoping review and applications on Belgian COVID-19 data. BMC Infect Dis.

[10] Antonelli M, Pujol JC, Spector TD, Ourselin S, Steves CJ (2022). Risk of long COVID associated with delta versus omicron variants of SARS-CoV-2. The Lancet.

[11] Hedberg P, Nauclér P. Post COVID-19 condition after SARS-CoV-2 Infections during the omicron surge compared with the delta, alpha, and wild-type periods in Stockholm, Sweden. J Infect Dis. 2023:jiad382. 10.1093/infdis/jiad382 PMC10786247 37665981

[12] Magnusson K, Kristoffersen DT, Dell’Isola A, Kiadaliri A, Turkiewicz A, Runhaar J (2022). Post-covid medical complaints following Infection with SARS-CoV-2 omicron vs Delta variants. Nat Commun.

[13] Ballouz T, Menges D, Kaufmann M, Amati R, Frei A, von Wyl V (2023). Post COVID-19 condition after Wildtype, Delta, and Omicron SARS-CoV-2 Infection and prior vaccination: pooled analysis of two population-based cohorts. PLoS ONE.

[14] Smith P, Proesmans K, Van Cauteren D, Demarest S, Drieskens S, De Pauw R (2022). Post COVID-19 condition and its physical, mental and social implications: protocol of a 2-year longitudinal cohort study in the Belgian adult population. Archives of Public Health.

[15] Touma M (2020). COVID-19: molecular diagnostics overview. J Mol Med.

[16] Proesmans K, Hancart S, Braeye T, Klamer S, Robesyn E, Djiena A (2022). COVID-19 contact tracing in Belgium: main indicators and performance, January - September 2021. Arch Public Health.

[17] National Institute for Health and Care Excellence (2020). Clinical guidelines. COVID-19 rapid guideline: managing the long-term effects of COVID-19.

[18] Fernández-de-Las-Peñas C, Palacios-Ceña D, Gómez-Mayordomo V, Cuadrado ML, Florencio LL (2021). Defining post-COVID symptoms (post-acute COVID, long COVID, persistent post-COVID): an integrative classification. Int J Environ Res Public Health.

[19] Sciensano, Belgium. COVID-19 Epidemiological Situation [Available from: https://datastudio.google.com/embed/u/0/reporting/c14a5cfc-cab7-4812-848c-0369173148ab/page/urrUC.

[20] Sciensano, RECOMMANDATIONS POUR LA SÉLECTION D’ÉCHANTILLONS EN VUE DU SÉQUENÇAGE DU GÉNOME. COMPLET 2021 [Available from: https://covid-19.sciensano.be/sites/default/files/Covid19/20210125_RAG_Selection%20of%20samples%20for%20sequencing_FR.pdf.

[21] (ECDC) ECfDPaC. Evaluation of the SARS-CoV-2 testing policy in Belgium from June to December 2021 2022 [Available from: https://www.ecdc.europa.eu/sites/default/files/documents/covid-19-evaluation-SARS-CoV-2-testing-policy-Belgium-Feb-2022.pdf.

[22] Braeye T, Catteau L, Brondeel R, van Loenhout JAF, Proesmans K, Cornelissen L (2022). Vaccine effectiveness against onward transmission of SARS-CoV2-infection by variant of concern and time since vaccination, Belgian contact tracing, 2021. Vaccine.

[23] R Core Team. R: A language and environment for statistical Computing. R Foundation for Statistical Computing, Vienna, Austria [Available from: https://www.R-project.org/.

[24] Cole SR, Zivich PN, Edwards JK, Ross RK, Shook-Sa BE, Price JT (2023). Missing Outcome data in epidemiologic studies. Am J Epidemiol.

[25] Grint DJ, Wing K, Houlihan C, Gibbs HP, Evans SJW, Williamson E (2022). Severity of severe Acute Respiratory System Coronavirus 2 (SARS-CoV-2) alpha variant (B.1.1.7) in England. Clin Infect Dis.

[26] Luo CH, Morris CP, Sachithanandham J, Amadi A, Gaston D, Li M et al. Infection with the SARS-CoV-2 delta variant is associated with higher infectious virus loads compared to the alpha variant in both unvaccinated and vaccinated individuals. MedRxiv. 2021:2021.08. 15.21262077. 10.1093/cid/ciab986 PMC8903351 34922338

[27] Canas LS, Molteni E, Deng J, Sudre CH, Murray B, Kerfoot E et al. Profiling post-COVID-19 condition across different variants of SARS-CoV-2: a prospective longitudinal study in unvaccinated wild-type, unvaccinated alpha-variant, and vaccinated delta-variant populations. Lancet Digit Health. 2023. 10.1016/S2589-7500(23)00056-0 PMC10187990 37202336

[28] Vallée A (2021). Dysautonomia and implications for Anosmia in Long COVID-19 Disease. J Clin Med.

[29] Fernández-de-Las-Peñas C, Cancela-Cilleruelo I, Rodríguez-Jiménez J, Gómez-Mayordomo V, Pellicer-Valero OJ, Martín-Guerrero JD (2022). Associated-onset symptoms and post-COVID-19 symptoms in hospitalized COVID-19 survivors infected with Wuhan, Alpha or Delta SARS-CoV-2 variant. Pathogens.

[30] Association WM (2001). World Medical Association Declaration of Helsinki. Ethical principles for medical research involving human subjects. Bull World Health Organ.

