# Supplementary material for: Association between SARS-CoV-2 variants and post COVID-19 condition: findings from a longitudinal cohort study in the Belgian adult population
Source: BMC Infect Dis. 2023 Nov 8;23:774. doi: 10.1186/s12879-023-08787-8 (PMC10634063; doi:10.1186/s12879-023-08787-8)
Supplement: Supplementary file 1 — Supplementary Material 1 [file 12879_2023_8787_MOESM1_ESM.docx]

***Supplementary table 1. Sensitivity analysis, multivariable model of PCC status with the threshold of the dominant variant is 70% and 90%***

| **Characteristic** | **Multivariable model*** | | |
| --- | --- | --- | --- |
|  | **Odds Ratio** | **95% Confidence Interval** | **p-value** |
| **Threshold of the dominant variant is 70%** |  |  |  |
| **Variant** |  |  |  |
| Omicron | REF | REF | REF |
| Alpha | 1.6 | 1.35, 1.91 | <0.001 |
| Delta | 1.74 | 1.55, 1.94 | <0.001 |
| **Threshold of the dominant variant is 90%** |  |  |  |
| **Variant** |  |  |  |
| Omicron  Alpha | REF  No cases | REF  No cases | REF  No cases |
| Delta | 1.73 | 1.55, 1.94 | <0.001 |
| * Adjusted for sex, age, education, Body Mass Index (BMI), having a chronic disease, number of Covid-19 acute symptoms, COVID-19 vaccination status, hospitalization status | | | |
