# Supplementary material for: Association between SARS-CoV-2 variants and post COVID-19 condition: findings from a longitudinal cohort study in the Belgian adult population
Source: BMC Infect Dis. 2023 Nov 8;23:774. doi: 10.1186/s12879-023-08787-8 (PMC10634063; doi:10.1186/s12879-023-08787-8)
Supplement: Supplementary file 2 — Supplementary Material 2 [file 12879_2023_8787_MOESM2_ESM.docx]

***Supplementary table 2.*** ***General characteristics of study excluded and included study population***

| **Characteristic** | **Total cases in original data** | **Excluded cases** | **Included cases** | **p-value** |
| --- | --- | --- | --- | --- |
|  | **N = 9199** | **N = 961** | **N= 8238 (89.23%)** |  |
| PCC |  |  |  |  |
| Yes | 4,188 (45.5%) | 479 (49.8%) | 3,709 (45.0%) | 0.005 |
| No | 5,011 (54.6%) | 482 (50.2%) | 4,529 (55.0%) |  |
