# Supplementary material for: Association between SARS-CoV-2 variants and post COVID-19 condition: findings from a longitudinal cohort study in the Belgian adult population
Source: BMC Infect Dis. 2023 Nov 8;23:774. doi: 10.1186/s12879-023-08787-8 (PMC10634063; doi:10.1186/s12879-023-08787-8)
Supplement: Supplementary file 3 — Supplementary Material 3 [file 12879_2023_8787_MOESM3_ESM.docx]

***Supplementary table 3.*** ***General characteristics of included study population and Joint association Variant and Vaccination***

| **Joint association Variant and Vaccination** | **Included cases** | |
| --- | --- | --- |
|  | **n= 8238** | **%** |
| Vaccinated omicron | 3054 | 37.07 |
| Vaccinated alpha | 1038 | 12.60 |
| Vaccinated delta | 3536 | 42.92 |
| Non vaccinated omicron | 87 | 1.06 |
| Non vaccinated alpha | 82 | 1.00 |
| Non vaccinated delta | 314 | 3.81 |
| Missing data | 127 | 1.54 |
