# Supplementary material for: Association between SARS-CoV-2 variants and post COVID-19 condition: findings from a longitudinal cohort study in the Belgian adult population
Source: BMC Infect Dis. 2023 Nov 8;23:774. doi: 10.1186/s12879-023-08787-8 (PMC10634063; doi:10.1186/s12879-023-08787-8)
Supplement: Supplementary file 4 — Supplementary Material 4 [file 12879_2023_8787_MOESM4_ESM.docx]

***Supplementary table 4. Sensitivity analysis, comparison of multivariable model of PCC status with separate association vs join association with vaccination and variants***

| **Characteristic** | **Multivariable with joint association** | | | **Multivariable without joint association** | | |
| --- | --- | --- | --- | --- | --- | --- |
|  | **Odds Ratio** | **95% Confidence Interval** | **p-value** | **Odds Ratio** | **95% Confidence Interval** | **p-value** |
| Joint association Variant and Vaccination |  |  |  |  |  |  |
| Vaccinated omicron | ___ | ___ | ___ |  |  |  |
| Vaccinated alpha | 1.81 | 1.54, 2.13 | <0.001 |  |  |  |
| Vaccinated delta | 1.76 | 1.58, 1.97 | <0.001 |  |  |  |
| Non vaccinated omicron | 0.67 | 0.39, 1.11 | 0.13 |  |  |  |
| Non vaccinated alpha | 0.80 | 0.47, 1.33 | 0.4 |  |  |  |
| Non vaccinated delta | 1.14 | 0.87, 1.50 | 0.3 |  |  |  |
| Variant |  |  |  |  |  |  |
| Omicron |  |  |  | ___ | ___ | ___ |
| Alpha |  |  |  | 1.61 | 1.33, 1.96 | <0.001 |
| Delta |  |  |  | 1.73 | 1.54, 1.93 | <0.001 |
| Gender |  |  |  |  |  |  |
| Male | ___ | ___ | ___ | ___ | ___ | ___ |
| Female | 1.57 | 1.41, 1.75 | <0.001 | 1.57 | 1.41, 1.75 | <0.001 |
| Age |  |  |  |  |  |  |
| 18-25 | ___ | ___ | ___ | ___ | ___ | ___ |
| 26-45 | 1.00 | 0.84, 1.20 | >0.9 | 1.01 | 0.84, 1.21 | >0.9 |
| 46-65 | 0.95 | 0.79, 1.15 | 0.6 | 0.96 | 0.80, 1.16 | 0.7 |
| 66+ | 0.66 | 0.50, 0.86 | 0.002 | 0.66 | 0.50, 0.87 | 0.003 |
| Education |  |  |  |  |  |  |
| Secondary school or below | ___ | ___ | ___ | ___ | ___ | ___ |
| Higher education | 0.77 | 0.69, 0.86 | <0.001 | 0.77 | 0.69, 0.86 | <0.001 |
| Body Mass Index (BMI) |  |  |  |  |  |  |
| Normal (BMI 18.5-24.9) | ___ | ___ | ___ | ___ | ___ | ___ |
| Overweight (BMI 25.0-29.9) | 1.12 | 1.00, 1.25 | 0.052 | 1.12 | 1.00, 1.25 | 0.050 |
| Obesity (BMI 30.0+) | 1.38 | 1.20, 1.58 | <0.001 | 1.38 | 1.21, 1.58 | <0.001 |
| Having chronic disease |  |  |  |  |  |  |
| No | ___ | ___ | ___ | ___ | ___ | ___ |
| Yes | 1.62 | 1.33, 1.96 | <0.001 | 1.61 | 1.33, 1.96 | <0.001 |
| Number of Covid-19 symptoms at baseline |  |  |  |  |  |  |
| None | ___ | ___ | ___ | ___ | ___ | ___ |
| 1-4 | 1.86 | 1.41, 2.49 | <0.001 | 1.86 | 1.41, 2.49 | <0.001 |
| 5-8 | 2.95 | 2.24, 3.92 | <0.001 | 2.94 | 2.24, 3.91 | <0.001 |
| >8 | 6.01 | 4.54, 8.04 | <0.001 | 5.99 | 4.53, 8.02 | <0.001 |
| COVID-19 vaccination status |  |  |  |  |  |  |
| None |  |  |  | ___ | ___ | ___ |
| Partial |  |  |  | 1.74 | 1.06, 2.88 | 0.030 |
| Complete primary schedule |  |  |  | 1.74 | 1.38, 2.20 | <0.001 |
| Complete primary schedule and booster |  |  |  | 1.57 | 1.25, 1.96 | <0.001 |
| Hospitalization |  |  |  |  |  |  |
| No | ___ | ___ | ___ | ___ | ___ | ___ |
| Yes | 2.26 | 1.36, 3.87 | 0.002 | 2.25 | 1.35, 3.84 | 0.002 |
|  | | | | | | |
